# Telephone Deafness

**Published:** 1918-04

**Authors:** George Cott

**Affiliations:** Buffalo, N. Y.


### BUFFALO MEDICAL JOURNAL

Yearly Volume 73. APRIL, 1918. Number 9

### ORIGINAL ARTICLES

**The right is reserved to decline papers not dealing with practical medical and surgical subjects, and such as might offend or fail to interest readers. Contributors are solely responsible for opinions, methods of expression and revision of proof.**

### Telephone Deafness.

DR. GEORGE COTT, Captain M. R. C., Buffalo, N. Y.

A number of instances came to my notice in recent years in which people complained of impaired hearing due to shock or other causes when they put the telephone receiver against the auricle. I did not credit the complaints very much at the time but repeated similar experiences have given it a more serious aspect. Still the pathology was not clear to me until the telephone company referred several cases for examination as to the reality of conditions complained of by those seeking damages for the production of sudden deafness. Even after examination and definite conclusions that actual deafness existed, several of my colleagues contended that the particular individuals concerned were hysterical. I could not be convinced that they were not really deaf. I examined each one very carefully, noted the findings and then without making a report to the company made a second and third appointment in each case and comparing results at each sitting; I always found them the same. In each case the patient did not complain of difficult hearing before the shock was experienced.

The history stated that when putting the receiver to the ear they suddenly heard a loud high pitched sound then could not hear another thing. The condition remained without improvement for several weeks or until they were brought to my notice. Even though the cases were held to be hysterical it would have been hard to convince a jury that these people were not permanently deaf or that deafness was feigned. If such people were examined in court with all the different methods employed by the otologist to detect malingering they could not be trapped. I concluded properly that the auditory nerve was suffering from shock which in all probability left the rest of the labyrinth intact. It is assumed when one of the six labyrinthin branches was impaired the entire 8th nerve outside of the brain suffered likewise. That is probably true. On the other hand the cochlea being the youngest in the formation is more affected and is not so ready to recover. If the vestibular apparatus were tested shortly after the injury no doubt we would find impaired function in the static as well as in the auditory portions of the nerve. If the assumption, that impairment of one branch means involvement of all is true we ought to find in case of auditory nerve affection some signa of involvement of the vestibular portion also. No patient with hysteria can evade a positive reaction of the static portion even though the auditory branch seems dead. In such cases we would be apt to produce hyper-activity of the static labyrinth either in eye movements or vertigo or dizziness; in functional as in hysteria both would produce these manifestations. Such patients could then easily become seasick or carsick, an experience which they did not have before.

But again assuming that impairment of one branch of the auditory bundle meant involvement of all branches, may it not be possible that the injury to the cochlea was carried from the end organ to the brain itself along the auditory path only, especially if there were some vulnerable point in the medulla trapezoid body or the very sensitive acoustic striae in the floor of the 4th ventricle, which injury could become permanent. In that case the static nerve being less influenced than the cochlear could more readily recover and eventually leave no trace behind. In order to make this clear let us trace the six branches to the brain: the cochlear branch, one from the saccule, another from the utricle, the horizontal, the anterior and the posterior verticle all meet in the internal auditory canal and form the bundle of the 8th nerve for a very short space when they enter the medulla oblongata; here again they divide into six branches each following its own course in separate and distinct paths.

The horizonal semi-circular canal nerve divides in Deiter's neucleus in the walls of the 4th ventricle, one branch going through the triangular neucleus to the posterior longitudinal bundle through which eye movements are produced and the other branch passing through the juxtarestiform body in the inferior cerebellar peduncle to the cerebellar nuclei.

The verticle canal branches pass through the medulla into the pons and in its upper portion divides; one branch goes also to the posterior longitudinal bundle through which nystagmus is produced and the other through the middle peduncle of the cerebellum. The saccule and utricle acting with the other static nerves govern antro-posterior and lateral motion.

Now the auditory or the portio dura of the 8th nerve has its own path. In the medulla it divides into an anterior and posterior branch; the anterior goes to the trapezoid body on its own side then passes over to the trapezoid body of the opposite side, then to the posterior corpora quadrigemina, the median geniculate body to the subthal-amic region where by association fibers the path leads to the posterior portion of the first temporal convolution. The posterior branch passes along the outer border of the medulla and the inferior cerebellar peduncle to the floor of the 4th ventricle, then after leaving the 4th ventricle it comes forward to enter the trapezoid body. The vestibular paths leading from the cerebellum to the brain cortex is not germane to the subject. Now if we can have lesions, and we do have them, affecting the different branches of the static nerve why is it not reasonable to suppose the auditory branches may likewise be influenced separately anywhere along its path in the brain stem or higher up, to a point of degeneration perhaps beyond recovery.

This would seem to have been so in these telephone cases, always provided they do not eventually recover which I very much doubt. It is just as likely that there was a vulnerable spot along the nerve path or rather in the nerve branch itself, and if recovery after a lengthy period does not take place the diagnosis of nerve deafness is absolute. Of my own experience I should judge that recovery takes place only to a certain degree but never complete.

Finally how is this telephone deafness caused? Any interference with the line while one is listening may jar the nerve suddenly, causing a high pitched sound followed by prolonged tinnitus with immediate deafness either partial or total. After some weeks the symptoms somewhat ameliorate.

The great point in which the public is most interested is how can telephone deafness be avoided. The company is absolutely helpless and at the mercy of the victim. The company cannot avoid the accident but the telephone users can. The accident occurs in one way only and that is when the receiver is held in contact with the ear so that no sound can interfere when listening to a speaker at the other end of the line. That is a common way to use the receiver when there is much noise around. If the user of the 'phone applies the receiver so that one part only touches the ear or the side of the head leaving a space between the ear and the rest of the instrument for free circulation of air it is impossible to transmit a shock to the nerve no matter how much disturbance there is on the line. This is actual experience. I would therefore advise the public rather to bear with a little noise than to run chances of suffering a degree of deafness which is embarrassing to you and to society in general.

My attention was first attracted by a case referred to me by the Telephone Company with a history of complete deafness following a shock while holding a receiver too close to the ear; this was accompanied by noises as the patient said, similar to the roaring of Niagara Falls. Patient was very nervous, also complained of pain since shock, like toothache. The noise in the ear is now like a steam engine.

The vestibular test now after 16 months shows improvement of function. Douching requiring 60 seconds to beginning of nystagmus of verticle canals although turning is normal 25 and 23 seconds. Deafness in the injured ear is still complete.

The second case, a very nervous female, aged 40, with apparently perfect hearing, received a shock in her left ear while using the telephone. She became totally deaf in both ears that night but the uninjured ear cleared up again within a week, the other remained deaf. She has a continuous rumbling noise. Her nervousness has cleared up in a great measure but one year has now elapsed, and she remains totally deaf in the injured ear.

The third case was a physician who four months ago received a similar shock which caused almost complete deafness in the left ear accompanied by a high pitched noise, leaving constant tinnitus ever since. For four weeks he could not use the telephone at all but recently hearing has improved so that he can again use the 'phone but hearing is still very dull and the prospect of ultimate recovery is not at all bright.

Since writing this article I have been told of other similar cases by other physicians. If the company would put up directions how to use the receiver perhaps it would do much towards preventing future occurrences and thus save money and a law suit.

---

**State Civil Service Commission,** Albany, will hold, in the early part of April, examinations for Superintendent of the County Tuberculosis Hospitals of Niagara and Steuben Counties. Salaries range from $1500 to $3000.

